# Unbound cloxacillin plasma concentrations in relation to toxicity and renal function: protocol for a prospective, observational clinical trial in a real-world Staphylococcus aureus bacteraemia population

**DOI:** 10.1136/bmjopen-2025-109745

**Published:** 2026-04-01

**Authors:** Tobias Damgaard, Thomas Hellborg, Anders Larsson, Erik Eliasson, Jonas Bonnedahl, Elisabet I Nielsen, Thomas Schön, Anita Hällgren

**Affiliations:** 1 Department of Biomedical and Clinical Sciences, Linköping University, Linköping, Sweden; 2 Pharmaceutical Department, Region Kalmar län, Kalmar, Sweden; 3 Department of Infectious Diseases, Region Kalmar län, Kalmar, Sweden; 4 Department of Medical Sciences, Uppsala University, Uppsala, Sweden; 5 Department of Clinical Chemistry, Uppsala University Hospital, Uppsala, Sweden; 6 Department of Laboratory Medicine, Karolinska Institutet, Stockholm, Sweden; 7 Division of Clinical Pharmacology, Karolinska University Hospital, Stockholm, Sweden; 8 Department of Pharmacy, Uppsala University, Uppsala, Sweden; 9 Department of Infectious Diseases, Linköping University Hospital, Linköping, Sweden

**Keywords:** Antibiotics, INFECTIOUS DISEASES, NEPHROLOGY, Observational Study, Toxicity, NEUROLOGY

## Abstract

**Introduction:**

*Staphylococcus aureus* bacteraemia (SAB) is a common and severe infection, with a 90-day mortality of 24%–32%. Cloxacillin is regarded as a first-line antibiotic treatment in SAB in Sweden. However, exposure to cloxacillin in real-world hospitalised patients with SAB, most of whom are elderly patients treated outside the intensive care unit, is not well described. There are also limited data on the role of unbound cloxacillin exposure in relation to renal function or drug-induced toxicity.

**Methods and analysis:**

This multicentre, prospective, observational clinical trial will include 95 adult patients with methicillin-susceptible *S. aureus* bacteraemia, treated with cloxacillin. Patients with endocarditis, polymicrobial bacteraemia or those considered unsuitable for cloxacillin treatment are excluded. Trough and peak total and unbound cloxacillin concentrations will be measured at steady state at days 2 and 7. Blood cultures will be obtained at days 2, 3, 4 and 7 to assess time to negative culture. Renal function will be assessed daily for plasma creatinine and at days 1 and 6 for cystatin C and for 12-hour urine creatinine clearance. In a novel approach to detecting nephrotoxicity, renal tubular damage biomarkers will be measured at days 1 and 6 (KIM-1, N-acetyl-β-D-glucosaminidase, neutrophil gelatinase-associated lipocalin, urine cystatin C, alpha-1-microglobulin). Detection of neurologic symptoms such as confusion, tremor, hallucinations and convulsions, as well as consciousness, will be monitored daily using a structured evaluation form.

We aim to investigate to which extent target attainment (100% of the dosing interval during which the free (unbound) drug concentration exceeds the minimum inhibitory concentration) is achieved with standard dosing of cloxacillin in a real-world cohort of hospitalised patients with SAB, and whether initial renal function can predict who is at risk for underdosing or overdosing. We will also explore whether neurological or renal damage is prevalent and associated with cloxacillin levels.

**Ethics and dissemination:**

Ethics approval has been granted by the Swedish Ethical Review Authority (EUCT 2023-505148-20-00) as part of a low-intervention clinical trial approval according to EU regulation 536/2014. Results will be disseminated in a peer-reviewed journal and at academic conferences.

**Trial registration number:**

EUCT 2023-505148-20-00.

**STRENGTHS AND LIMITATIONS OF THIS STUDY**

- Investigating cloxacillin treatment in a real-world, elderly *Staphylococcus aureus* bacteraemia population.
- Measuring unbound cloxacillin concentrations and assessing renal toxicity with several established and novel markers.
- Due to a limited sample size, any association between concentration and toxicity should be considered exploratory.

## Introduction

### Background

*Staphylococcus aureus* bacteraemia (SAB) is a severe infection with a reported prevalence of 9.3–65 cases/100 000/year and a 30-day and 90-day mortality rate ranging from 14%–23% and 24%–32%, respectively.^1^,^3^ Isoxazolyl penicillins, such as cloxacillin, are considered a first-line therapy when treating methicillin-susceptible SAB.^4^ Standard dosage of intravenous cloxacillin for SAB varies depending on infection severity and local dosing recommendations.^5 6^ Currently, a dose of 2 g every 6–8 hours is recommended for SAB without endocarditis in Sweden.^7 8^

In general, beta-lactam antibiotics show a time-dependent efficacy, where an insufficient percentage of the dosing interval during which the unbound (free) antibiotic concentration remains above the minimum inhibitory concentration (MIC) (fT>MIC) increases the risk of clinical failure and resistance development.^9 10^ For severe infections, it is recommended that unbound (free) plasma concentration remains above the MIC of the pathogen during therapy (100% fT>MIC).^11^ Cloxacillin is highly protein bound, like other isoxazolyl penicillins (eg, dicloxacillin and flucloxacillin), with an unbound fraction of 6%–8% based on data from the manufacturer.^7 12^ There are limited data on whether recommended dosing of intermittently given cloxacillin is sufficient to reach the recommended target in real-world patients with SAB. Since flucloxacillin protein binding has shown a variability between 2% and 72% in critically ill patients,^13^ an individualisation of cloxacillin dosing may be warranted.

Most therapeutic drug monitoring studies of cloxacillin involve critically ill patients with SAB in the intensive care unit (ICU) or patients with infective endocarditis.^14^,^16^ However, the typical patient with SAB is older (66–75 years) without endocarditis and is treated outside of the ICU.^17^,^20^ In younger patients outside the ICU, a small study in which total concentrations were measured suggests a low probability of achieving 100% fT>MIC with intermittent cloxacillin dosing regimens.^21^ However, these results may not be applicable to an elderly population, among whom an increased prevalence of chronic kidney disease can be anticipated,^22^ since augmented renal clearance is a risk factor for insufficient exposure of beta-lactam antibiotics.^23^ Estimation of renal function has shown potential in identifying insufficient exposure among ICU patients for some low-to-medium protein bound beta-lactam antibiotics,^24^ but the relevance for highly protein bound antibiotics among patients with SAB outside of the ICU is unknown.

Acute kidney injury (AKI) is common for patients with SAB, with a reported incidence between 17% and 43%, and is associated with an increased 30-day mortality.^25^,^27^ Although AKI has been considered to be caused by the infection per se, treatment with isoxazolyl penicillins has also been associated with an increased risk of nephrotoxicity.^28^ There are limited data on whether the increased risk of isoxazolyl penicillin-induced AKI is concentration-dependent, but small studies suggest that about 57%–71% of patients with manifest AKI had a cloxacillin concentration above 50 mg/L.^29 30^ The reported median time of AKI recovery after cloxacillin cessation is 5 days, which implies that early detection of AKI is beneficial for timely dose adjustment.^29^ However, an increase of serum creatinine as a routine marker for kidney injury is rarely detected until 1–2 days after AKI onset. AKI onset usually occurs within days after therapy initiation,^29^ suggesting the development of acute tubular necrosis.^29 31^ Biomarkers of renal tubular injury may have the potential to detect AKI at an earlier stage than serum creatinine,^32^ but their diagnostic potential has not been widely evaluated in patients treated with beta-lactam antibiotics.^33^

Recent findings suggest that high concentrations of beta-lactam antibiotics may be associated with neurologic symptoms, including persistent coma and delirium, resulting in longer hospital stays.^16 34 35^ A small study reported that neurologic symptoms in critically ill patients improved for all patients after dose reduction of cloxacillin.^16^ It is therefore important to identify whether neurological symptoms may be associated with antibiotic concentration levels, including non-ICU patients with SAB. We plan to conduct a prospective observational study in a real-world, hospitalised population with SAB. The study is approved as a low-interventional clinical trial registered in CTIS (Trial registration number: EUCT 2023-505148-20-00).

We aim to investigate to which extent target attainment (100% fT>MIC) is achieved with standard dosing of cloxacillin in a real-world cohort of hospitalised patients with SAB, and whether initial renal function can predict who is at risk for underdosing or overdosing. Furthermore, we will explore whether renal- or neurotoxicity may be associated with unbound cloxacillin concentrations, including the potential for novel renal tubular damage biomarkers to identify beta-lactam induced AKI. Lastly, we will survey the trial participants’ experience of taking part in the trial.

## Methods and analysis 

### Trial design

The KLOX-SAB study is a multicentre, prospective, observational clinical trial investigating standard care treatment with cloxacillin for patients with SAB in southeast Sweden. The trial is registered as low-interventional and abides by Good Clinical Practice standards.^36^

### Trial population

The trial population will consist of adult patients with a blood culture with growth of *Staphylococcus aureus,* obtained no more than 72 hours prior to inclusion. The participant is treated with cloxacillin therapy started at the time of, or no more than 72 hours prior to, inclusion, and has given informed consent. Inclusion and exclusion criteria are found in table 1. Patients will be treated with standard-of-care according to the treating physician, normally intravenous cloxacillin 2 g every 6–8 hours for at least 7 days.

**Table 1:** Inclusion and exclusion criteria

Inclusion criteria	Exclusion criteria
Positive blood culture with *Staphylococcus aureus* obtained no more than 72 hours before inclusion	*S. aureus* not susceptible (S) to cloxacillin
≥18 years of age	Polymicrobial bacteraemia or concomitant infection which demands treatment with an antibiotic with broader spectrum than cloxacillin.
Treatment with cloxacillin, started at inclusion or no more than 72 hours before inclusion	Endocarditis or other intracardial infections detected by echocardiography.
Informed oral and written consent	Serious allergic reaction to cloxacillin, other penicillins or cephalosporins.
	Any contraindication for cloxacillin treatment or trial participation as judged by the treating physician.
	Moribund; when a cure is not deemed achievable with active treatment and aim of care is to give end-of-life or best supportive care
	Breastfeeding and not able or willing to interrupt breastfeeding
	Unconsciousness, not willing to participate or assessed as having difficulties understanding the significance of trial participation due to mental disability or language barriers.
	Participation in a clinical trial 30 days prior to initiation
	Prior participation in the current trial

Cloxacillin susceptibility is assessed using clinical break-points from 2025 by the European Committee on Antimicrobial Susceptibility Testing (EUCAST).^5^

Participants with no available echocardiography at the time of inclusion may still be recruited. If endocarditis is detected with echocardiography later during the trial, the participant will be excluded.

### Recruitment strategy

Participants admitted to the University Hospital in Linköping, Vrinnevi Hospital in Norrköping or Kalmar County Hospital and who are deemed eligible will be recruited. The trial aims to recruit 95 patients. A clinical routine has been established at each site previous to the trial, where each positive SAB finding initiates an infectious specialist consultation, including bedside assessment when possible.

### Trial procedures

The trial duration for each participant will be 30 days. Initial blood and urine sampling will occur during the first 7 in-hospital days, and outcome data will be gathered on day 30 from the medical record (table 2).

Each positive blood culture will be reported to an infection specialist by direct contact from the microbiology lab at each site. Eligibility of trial participation will be assessed, where eligible patients are informed about and asked to participate in the trial by a doctor or nurse associated with the trial. After written informed consent is obtained, participant characteristics are registered (gender, age, weight, height). Comorbidity will be estimated using the Charlson comorbidity index.^37^ Infection severity will be estimated via the National Early Warning Score 2.^38^ Current medications and dosing at time of inclusion will be recorded as will any addition, withdrawal or dosing adjustments during the trial duration. Prevalence of neurologic symptoms will be registered with occurrence of tremor, hallucination, convulsions or confusion corresponding to ≥2 points measured by Nursing Delirium Screening Scale,^39^ as well as consciousness level assessed using the Reaction Level Scale-85.^40^ Blood cloxacillin concentrations will be sampled prior to administration (trough) as well as within 30 min after end of cloxacillin infusion (peak) (table 2). Additional measurements of plasma liver and renal biomarkers (alanine aminotransferase (ALT), cystatin C, creatinine and albumin) and blood cultures (aerobic+anaerobic) will be sampled at the same time as clinical sampling or trough level sampling.

**Table 2:** Trial procedures

Procedures	Day							
	1	2	3	4	5	6	7	30 (28–35)
Inclusion/exclusion criteria eligibility	√							
Informed consent	√							
Demographic and clinical data, comorbidity, current medication, baseline symptoms CNS	√							
Neurological symptoms, NEWS-2, medication and dosing adjustments	√	√	√	√	√	√	√	
Plasma samples: ALT, cystatin C, albumin.	√					√		
Plasma creatinine, eGFR estimation	√	√	√	√	√	√	√	
Urine sample for renal damage marker measurement and initiation of 12 hours urine sampling for creatinine clearance measurement	√					√		
Plasma samples for cloxacillin-concentration measurement (peak and trough) and urine creatinine measurement with creatinine clearance calculation		√					√	
Blood culture		√	√	√			√	
Final diagnosis, duration of bacteraemia, mortality, complexity of infection								√
Serious adverse events (deaths)		√	√	√	√	√	√	√
End of trial								*√*

ALT, alanine aminotransferase; CNS, central nervous system; eGFR, estimated glomerular filtration rate; NEWS, National Early Warning Score.

Urine will be sampled for renal tubular damage biomarker measurements (cystatin C, kidney injury molecule 1 (KIM-1), N-acetyl-β-D-glucosaminidase (NAG), neutrophil gelatinase associated lipocalin (NGAL), alpha-1-microglobulin (A1M) and additional biomarkers associated with renal damage). A 12-hour urine collection will subsequently be initiated to measure creatinine clearance and total urine production (table 2). Total urine volume will be determined by weight and sampled for creatinine clearance analysis.

At the end of the trial participation, mortality, lost-to-follow-up, time to negative SAB blood culture, assessment of SAB complexity according to Infectious Diseases Society of America,^41^ and diagnosis codes will be collected from the medical record.

More details on the frequency of data and sample collection can be found in table 2.

### Selected cloxacillin PK/PD targets for efficacy and toxicity

Methicillin-susceptible *S. aureus* shows a cloxacillin MIC distribution epidemiological cut-off (ECOFF) breakpoint at 0.5 mg/L, based on wild-type distributions from the EUCAST.^42^ Thus, the exposure target in this trial is set to 100% fT>MIC_ECOFF_, maintaining an unbound concentration above 0.5 mg/L.^11^ Overexposure is defined as maintaining an unbound concentration above 5 mg/L (100% fT>10xMIC_ECOFF_) or a corresponding total concentration above 50 mg/L, which was found in >50% of patients with a manifest AKI.^29 30 43^ AKI will be assessed by the change of serum creatinine levels, as described in Kidney Disease: Improving Global Outcomes (KDIGO) criteria.^44^ Neurological symptoms such as persistent coma and delirium have been associated with increased total trough cloxacillin concentrations with a lower range of 100 mg/L.^16^

### Sample analysis

Plasma ALT, cystatin C, creatinine and albumin as well as urine A1M and urine creatinine clearance will be analysed using validated clinical routine methods at accredited hospital laboratories. Cloxacillin samples will be centrifuged (2000 g, 10 min) within 1 hour of sampling, aliquoted into 6×500 µL cryotubes and stored in −80°C until analysis. Total and free fraction of cloxacillin will be analysed at Karolinska University Laboratory (Stockholm, Sweden), using ultrafiltration and LC-MS/MS with validated methods available in clinical routine.^45^

Renal tubular damage biomarkers will be analysed from frozen urine at Academic Laboratory (Uppsala, Sweden), where KIM-1 and NGAL will be analysed using commercial sandwich ELISAs (R&D Systems, Bio-Techne, Dublin, Ireland), and urine cystatin C and NAG with a Mindray BS380 chemistry analyser (Mindray Medical International, Shenzhen, China). The reference intervals used will be <0.414 mg/L (cystatin C^46^) and <10 mg/L (A1M). As there is no internationally recognised reference material for KIM-1, NAG and NGAL, the standard interval will be set to two SD above the mean for each assay according to the manufacturer. Additional urine renal damage biomarkers may come to be analysed using validated methods.

On day 30 (28–35), a proportion of the trial subjects will be asked about their experience of the trial through a questionnaire or a brief, semistructured interview to provide feedback on their experience of participation in the trial.

### Trial duration

Trial recruitment was planned to start in January 2024, but was delayed until 15 July 2024. Recruitment finalisation was planned to 31 July 2026 but has been postponed to a preliminary date of 31 December 2026. An interim analysis will be conducted mid-term with the possibility of trial prolongation.

### Endpoints

Primary endpoints:

1. Proportion of patients with SAB reaching 100% ƒT>MIC_ECOFF_, that is, an unbound cloxacillin trough level of >0.5 mg/L at days 2 and 7 and its association with renal function.
2. Proportion of patients with SAB reaching an unbound cloxacillin trough level of >5 mg/L or total trough level of >50 mg/L at days 2 and 7 and its association with renal function.

Secondary endpoints:

- Prevalence of increased levels of urine markers for renal tubular damage (cystatin C, KIM-1, NAG, NGAL, A1M) compared with reference values, and its association with cloxacillin concentrations (peak and trough) at days 2 and 7.
- Prevalence of AKI based on creatinine, defined by KDIGO, measured daily between days 1 and 7.
- Onset of new symptoms from the central nervous system (ie, decreased consciousness, seizures, tremor, hallucinations or confusion), with day 1 regarded as baseline, measured daily on days 1–7, in relation to cloxacillin concentrations (peak and trough) at days 2 and 7.
- Descriptive characteristics of the SAB population, including sex, age, weight, comorbidity, medications, infection severity, complexity, duration and 30-day mortality.
- Levels of additional exploratory biomarkers in urine and plasma associated with nephrotoxicity or neurotoxicity.
- The trial participants’ experience taking part in the trial.

### Data management

Data will be recorded with an electronic Case Report Form (REDcap, V.14.0.22, Vanderbilt University, Nashville, Tennessee, USA), requiring two-step verification. Data will be registered by the primary investigator (PI) or trial nurses at each site or members of the trial group as delegated by the PI. All data will be stored on secured servers at Linköping University. A copy of the locked eCRF will also be printed on paper. All trial-associated documents will be stored at each site until completion of the trial and will afterwards be archived for at least 25 years after the trial’s completion.

### Hypothesis and sample size calculations

In this low-interventional clinical trial, sample size calculation is primarily needed for the analyses in relation to renal function.

We have previously shown that an eGFR cut-off of approximately 45 mL/min/1.73 m^2^ may be used to predict sufficient exposure in a study on beta-lactam antibiotic treatment within a population of patients in ICUs.^24^ We hypothesised that cloxacillin exposure is similar to other beta-lactam antibiotics when related to renal function. Cut-offs were selected from chronic kidney disease stages according to clinical applicability as well as superiority in sensitivity and specificity in detecting insufficient exposure for other beta-lactam antibiotics.^24^

Based on the literature and our own unpublished clinical data (median age for patients with SAB in Region Östergötland is 72 years), we hypothesised that the proportion with eGFR <45 mL/min/1.73 m^2^ in the trial population (including individuals with HD, estimated ratio 10%) will be approximately 35%–40%.^22 47^

Furthermore, we hypothesise that 90% of individuals with eGFR ≤45 mL/min/1.73 m^2^ will reach the target of 100% ƒT>MIC_ECOFF_ (ie, a trough unbound concentration of cloxacillin >0.5 mg/L). This assumption was based on a limited number of previous studies where total cloxacillin concentrations were measured and related to eGFR.^15 16^ Based on the same studies, we hypothesised that 50% of individuals with eGFR ≤45 mL/min/1.73 m^2^ will reach tentatively toxic trough concentrations (ie, total concentration >50 mg/L, or unbound concentration >5 mg/L). For patients≥45 mL/min/1.73 m^2^, we hypothesise that 50% will reach an unbound concentration of at least 0.5 mg/L and for patients >60 mL/min/1.73 m^2^, 20% will have >5 mg/L.^16^

To ensure sufficient sample size in each renal function group, we estimated that 38 individuals with eGFR ≤45 mL/min/1.73 m^2^ and 57 patients with eGFR above 45 mL/min/1.73 m^2^ are required to detect a difference in predefined exposure cut-offs between renal groups, with a power of 0.8 and alpha value of 0.05. Consequently, at least 95 individuals will need to be included.

If participants decide to withdraw prematurely before day 4, a new participant will be recruited. Data collected prior to the withdrawal will be used in the analysis.

When 45 participants have been recruited, an interim analysis of renal function distribution is performed to evaluate if the sample size estimation for each renal function group is feasible. Furthermore, it is evaluated whether missing data concerning primary outcomes or the rate of patients with renal replacement therapy is higher than expected. Recruitment progression is not dependent on the results of the interim analysis, but the total number of participants may be adjusted accordingly to ensure sufficient power in each renal function group.

### Statistical analysis

Continuous variables are expressed as mean±SD if normally distributed and as median and IQR if not. Categorical variables are expressed as numbers and percentages.

Primary endpoints are analysed with univariate logistic regression and receiver operating characteristics (ROC) curves. A multivariate logistic regression is performed including all univariate variables with a p<0.1. Results from the multivariate regression are expressed with OR, area under the curve and 95% CI. A p<0.05 will indicate statistical significance. Multiplicity due to interim analysis will be handled using the method suggested by Haybittle-Peto, where a p<0.001 is deemed significant for the interim analysis and <0.05 for the main analysis.^48^

Secondary endpoints are presented demographically and analysed with linear regression for continuous outcomes and logistic regression for dichotomous outcomes. Results should be considered exploratory.

Multiple imputation techniques will be performed if missing data is equal to less than 10% of the total number of data points for the variable.

### Patient and public involvement

Due to the real-world setting and observational character of the clinical trial, no patient has been involved in trial design development. However, to prepare for future studies, participants will be contacted 30 days after inclusion and asked to answer a short questionnaire to survey their experiences of taking part in the trial.

## Ethics and dissemination

The study has been approved as a low-intervention clinical trial according to EU regulation 536/2014, with approval number 2023-505148-20-00. Ethical approval has therefore been granted as part of the procedure.

This observational trial evaluates current standard of care. Cloxacillin concentration measurements will not be available for treating physicians. To minimise patient suffering, blood samples will be drawn at the same time as routine sampling when possible, and all medical record data will be transferred to the pseudonymised eCRF for further analysis. Serious adverse events, where an association with cloxacillin therapy is deemed likely, will be reported to the Swedish Medical Products Agency. Only death will be considered as a serious adverse event in this real-world, observational clinical trial.

Participants may withdraw at any time, and no further data will then be collected. All data collected before the withdrawal will be used in the analysis, which is stated in the informed consent given at the trial initiation.

Analyses will be performed using pseudonymised data. All results will be presented groupwise and one or more manuscripts will be submitted to peer-reviewed journals and presented at academic conferences. The trial protocol is registered in CTIS (EUCT: 2023-505148-20-00).

## References

[1] UK Health Security Agency (2023). MRSA, MSSA, gram-negative bacteraemia and CDI: 30-day all-cause mortality.

[2] Bell J, Fajardo Lubian A, Partridge S (2018). Australian Group on Antimicrobial Resistance (AGAR) Australian Gram-negative Surveillance Outcome Program (GnSOP) Bloodstream Infection Annual Report 2024. Commun Dis Intell (2018).

[3] Swets MC, Bakk Z, Westgeest AC (2024). Clinical Subphenotypes of Staphylococcus aureus Bacteremia. Clin Infect Dis.

[4] Loubet P, Burdet C, Vindrios W (2018). Cefazolin versus anti-staphylococcal penicillins for treatment of methicillin-susceptible Staphylococcus aureus bacteraemia: a narrative review. Clin Microbiol Infect.

[5] The European Committee on Antimicrobial Susceptibility Testing (EUCAST) (2025). Dosages used to define breakpoints. https://www.eucast.org/clinical_breakpoints.

[6] Tong SYC, Fowler VG, Skalla L (2025). Management of Staphylococcus aureus Bacteremia: A Review. JAMA.

[7] Läkemedelsindustriföreningen (LiF) (2024). FASS - summary of product characteristics. https://www.fass.se/LIF/startpage.

[8] (2024). Swedish Strategic Programme against antibiotic resistance (STRAMA). https://strama-nationell.infosynk.se/.

[9] Roberts JA, Paul SK, Akova M (2014). DALI: defining antibiotic levels in intensive care unit patients: are current β-lactam antibiotic doses sufficient for critically ill patients?. Clin Infect Dis.

[10] Sumi CD, Heffernan AJ, Lipman J (2019). What Antibiotic Exposures Are Required to Suppress the Emergence of Resistance for Gram-Negative Bacteria? A Systematic Review. *Clin Pharmacokinet*.

[11] Abdul-Aziz MH, Alffenaar J-WC, Bassetti M (2020). Antimicrobial therapeutic drug monitoring in critically ill adult patients: a Position Paper. Intensive Care Med.

[12] Røder BL, Frimodt-Møller N, Espersen F (1995). Dicloxacillin and flucloxacillin: pharmacokinetics, protein binding and serum bactericidal titers in healthy subjects after oral administration. Infection.

[13] Wallenburg E, Ter Heine R, de Lange DW (2021). High unbound flucloxacillin fraction in critically ill patients. J Antimicrob Chemother.

[14] Macheda G, El Helali N, Péan de Ponfilly G (2022). Impact of therapeutic drug monitoring of antibiotics in the management of infective endocarditis. Eur J Clin Microbiol Infect Dis.

[15] Verdier MC, Tribut O, Tattevin P (2011). Assessment of interindividual variability of plasma concentrations after administrazion of high doses of intravenous amoxicillin or cloxacillin in critically ill patients. J Chemother.

[16] Neuville M, El-Helali N, Magalhaes E (2017). Systematic overdosing of oxa- and cloxacillin in severe infections treated in ICU: risk factors and side effects. Ann Intensive Care.

[17] Ioannou P, Zacharioudaki M, Spentzouri D (2023). A Retrospective Study of *Staphylococcus aureus* Bacteremia in a Tertiary Hospital and Factors Associated with Mortality. Diagnostics (Basel).

[18] Hindy J-R, Quintero-Martinez JA, Lahr BD (2023). *Staphylococcus aureus* bacteraemia and mortality: a population-based study in Olmsted County, Minnesota, from 2006 to 2020. Infect Dis (Lond).

[19] Mathé P, Göpel S, Hornuss D (2023). Increasing numbers and complexity of Staphylococcus aureus bloodstream infection-14 years of prospective evaluation at a German tertiary care centre with multi-centre validation of findings. Clin Microbiol Infect.

[20] Strömdahl M, Hagman K, Hedman K (2025). Time to Staphylococcus aureus Blood Culture Positivity as a Risk Marker of Infective Endocarditis: A Retrospective Cohort Study. Clin Infect Dis.

[21] Courjon J, Garzaro M, Roger P-M (2020). A Population Pharmacokinetic Analysis of Continuous Infusion of Cloxacillin during *Staphylococcus aureus* Bone and Joint Infections. Antimicrob Agents Chemother.

[22] Werner KB, Elmståhl S, Christensson A (2014). Male sex and vascular risk factors affect cystatin C-derived renal function in older people without diabetes or overt vascular disease. Age Ageing.

[23] Abdulla A, Ewoldt TMJ, Purmer IM (2021). A narrative review of predictors for β-lactam antibiotic exposure during empirical treatment in critically ill patients. Expert Opin Drug Metab Toxicol.

[24] Damgaard T, Woksepp H, Brudin L (2024). Estimated glomerular filtration rate as a tool for early identification of patients with insufficient exposure to beta-lactam antibiotics in intensive care units. Infect Dis (Lond).

[25] Westgeest AC, Schippers EF, Delfos NM (2022). Acute kidney injury in Staphylococcus aureus bacteremia. Eur J Clin Microbiol Infect Dis.

[26] Buis DTP, van der Vaart TW, Mohan A (2024). Acute kidney injury in Staphylococcus aureus bacteraemia: a recurrent events analysis. Clin Microbiol Infect.

[27] Holmes NE, Robinson JO, van Hal SJ (2018). Morbidity from in-hospital complications is greater than treatment failure in patients with Staphylococcus aureus bacteraemia. BMC Infect Dis.

[28] Weis S, Kesselmeier M, Davis JS (2019). Cefazolin versus anti-staphylococcal penicillins for the treatment of patients with Staphylococcus aureus bacteraemia. Clin Microbiol Infect.

[29] Lavergne A, Vigneau C, Polard E (2018). Acute kidney injury during treatment with high-dose cloxacillin: a report of 23 cases and literature review. Int J Antimicrob Agents.

[30] Crochette R, Ravaiau C, Perez L (2021). Incidence and Risk Factors for Acute Kidney Injury during the Treatment of Methicillin-Sensitive *Staphylococcus aureus* Infections with Cloxacillin Based Antibiotic Regimens: A French Retrospective Study. J Clin Med.

[31] Tune BM (1997). Nephrotoxicity of beta-lactam antibiotics: mechanisms and strategies for prevention. Pediatr Nephrol.

[32] Bufkin KB, Karim ZA, Silva J (2024). Review of the limitations of current biomarkers in acute kidney injury clinical practices. SAGE Open Med.

[33] Campbell RE, Chen CH, Edelstein CL (2023). Overview of Antibiotic-Induced Nephrotoxicity. Kidney Int Rep.

[34] Zerbib Y, Gaulin C, Bodeau S (2023). Neurological burden and outcomes of excessive β-lactam serum concentrations of critically ill septic patients: a prospective cohort study. J Antimicrob Chemother.

[35] Venugopalan V, Casaus D, Kainz L (2023). Use of therapeutic drug monitoring to characterize cefepime-related neurotoxicity. Pharmacotherapy.

[36] Region Oestergoetland (2023). Kan skattning av njurfunktion (eGFR) förutsäga vilka patienter med S. aureus-bakteremi som behöver justerad dosering via monitorering av kloxacillinkoncentrationer i plasma (TDM)?. https://euclinicaltrials.eu/search-for-clinical-trials/?lang=en&EUCT=2023-505148-20-00.

[37] Ludvigsson JF, Appelros P, Askling J (2021). Adaptation of the Charlson Comorbidity Index for Register-Based Research in Sweden. Clin Epidemiol.

[38] Royal College of Physicians (2017). National early warning score (NEWS) 2: standardising the assessment of acute-illness severity in the NHS.

[39] Gaudreau J-D, Gagnon P, Harel F (2005). Fast, systematic, and continuous delirium assessment in hospitalized patients: the nursing delirium screening scale. J Pain Symptom Manage.

[40] Starmark JE, Stålhammar D, Holmgren E (1988). The Reaction Level Scale (RLS85). Manual and guidelines. Acta Neurochir (Wien).

[41] Liu C, Bayer A, Cosgrove SE (2011). Clinical practice guidelines by the infectious diseases society of america for the treatment of methicillin-resistant Staphylococcus aureus infections in adults and children. Clin Infect Dis.

[42] The European Committee on Antimicrobial Susceptibility Testing (EUCAST) (2024). Antimicrobial wild type distributions of microorganisms. https://mic.eucast.org/.

[43] Guilhaumou R, Benaboud S, Bennis Y (2019). Optimization of the treatment with beta-lactam antibiotics in critically ill patients-guidelines from the French Society of Pharmacology and Therapeutics (Société Française de Pharmacologie et Thérapeutique-SFPT) and the French Society of Anaesthesia and Intensive Care Medicine (Société Française d’Anesthésie et Réanimation-SFAR). Crit Care.

[44] Kidney Disease: Improving Gloval Outcomes (KDIGO) Acute Kidney Injury Work Group (2012). KDIGO Clinical Practice Guideline for Acute Kidney Injury. Kidney Inter Suppl.

[45] Beijer G, Clarin L, Östervall J (2023). Reproducible Quantification of Unbound Fractions of Four Beta-Lactam Antibiotics: Ultrafiltration Versus Microdialysis of Spiked Healthy Donor Plasma. Ther Drug Monit.

[46] Sohrabian A, Noraddin FH, Flodin M (2012). Particle enhanced turbidimetric immunoassay for the determination of urine cystatin C on Cobas c501. Clin Biochem.

[47] Hindy J-R, Quintero-Martinez JA, Lee AT (2022). Incidence Trends and Epidemiology of Staphylococcus aureus Bacteremia: A Systematic Review of Population-Based Studies. Cureus.

[48] Pocock S (1996). Clinical trials: a practical approach.

