# Supplementary material for: Unbound cloxacillin plasma concentrations in relation to toxicity and renal function: protocol for a prospective, observational clinical trial in a real-world Staphylococcus aureus bacteraemia population
Source: BMJ Open. 2026 Apr 1;16(4):e109745. doi: 10.1136/bmjopen-2025-109745 (PMC13052560; doi:10.1136/bmjopen-2025-109745)
Supplement: Supplementary file 1 [file bmjopen-2025-109745.reviewer_comments.pdf]

PEER REVIEW HISTORY

BMJ Open publishes all reviews undertaken for accepted manuscripts. Reviewers are asked to complete a checklist review form and are provided with free text boxes to elaborate on their assessment. These free text comments are reproduced below.

ARTICLE DETAILS

Title (Provisional)

Unbound cloxacillin plasma concentrations in relation to toxicity and renal function: protocol for a prospective, observational clinical trial in a real-world Staphylococcus aureus bacteremia population

Authors

Damgaard, Tobias; Hellborg, Thomas; Larsson, Anders; Eliasson, Erik; Bonnedahl, Jonas; Nielsen, Elisabet I; Schön, Thomas; Hällgren, Anita

---

VERSION 1 - REVIEW

**Reviewer**	**1**
**Name**	**Legg, Amy**
**Affiliation Department**	**Royal Brisbane and Women's Hospital, Pharmacy**
**Date**	**16-Dec-2025**
**COI**	**None**

Although the protocol is a worthwhile scientific investigation, some areas require clarification. I appreciate the efforts of the authors and commend them on this study.

Areas for review:

1. writing overall is poor and requires strengthening (some examples below)

2. need to explain the hypothesis being tested across the observational cohorts that resulted in formal sample size calculation. The more relevant clinical question would be concentrations that are associated with efficacy or toxicity? Why is this analysis not being undertaken?

3. How will you define what elevated urinary biomarker concentrations are?

Specific feedback:

The first sentence in abstract, introduction: Up to 30% of patients with ongoing antibiotic treatment do not survive Staphylococcus aureus bacteremia (SAB). This sentence doesn’t make sense to me, what type of patients are you describing here (ie those that survive to

step down or directed therapy)? This is higher than the usually quoted mortality rates for SAB.

Our aim is to describe the distribution of unbound cloxacillin exposure related to renal function and to explore if neurological- or renal damage is prevalent and associated with cloxacillin levels in real-world hospitalized SAB patients. – not sure distribution is the correct word here, maybe time-concentration profile, or temporal pharmacokinetics? Also, you aren't looking at the relationship of cloxacillin concentrations with kidney function in any endpoints. And this aim is only stated in the abstract.

In the introduction:

Renal function is a risk factor for insufficient exposure of beta-lactam antibiotics – specify augmented renal clearance is a risk factor for insufficient exposure of beta-lactam antibiotics.

Our aim is to describe the distribution of unbound cloxacillin exposure related to renal function and to explore if neurological- or renal damage is prevalent and associated with cloxacillin levels in real-world hospitalized SAB patients. – not sure distribution is the correct word here, maybe time-concentration profile, or temporal pharmacokinetics?

Oxacillin beta-lactam antibiotics – should be anti-staphylococcal penicillins?

Why would breastfeeding be interrupted?

Were pregnant women included?

Acute kidney injury is assessed by the change of serum creatinine levels, as described in the RIFLE criteria by Kidney Disease: Improving Global Outcomes (KDIGO) - RIFLE and KDIGO are separate.

As a secondary endpoint: Descriptive characteristics of the SAB population – only SAB patients treated with cloxacillin, will this be the majority of SAB patients in all hospitals?

VERSION 1 - AUTHOR RESPONSE

Reviewer: 1
Dr. Amy Legg, Royal Brisbane and Women's Hospital
Comments to the Author:
Although the protocol is a worthwhile scientific investigation, some areas require clarification. I appreciate the efforts of the authors and commend them on this study.
Areas for review:
1. writing overall is poor and requires strengthening (some examples below)

RESPONSE: We thank the reviewer for highlighting this and for the examples provided. We have thoroughly examined the manuscript with the help of a language reviewer before resubmission.

2. need to explain the hypothesis being tested across the observational cohorts that resulted in

formal sample size calculation. The more relevant clinical question would be concentrations that are associated with efficacy or toxicity? Why is this analysis not being undertaken?

RESPONSE: We agree with the reviewer that the ultimate question is "will the patient be cured without any harm made by the treatment". However, to study the relation between measured free concentrations and clinical effect (or harm), a much larger study would be necessary than what the study group judged as currently feasible (shown by the efforts of Roberts JA et al. in the DALI-study).

We have thus built on previous results and assumptions that for severe infections, an unbound (free) plasma concentration should remain at least above the MIC of the pathogen during the duration of therapy (100% fT>MIC) (as stated in Introduction page 2, lines 67-69) to optimize the chance for clinical success. In line with others, we have also found that eGFR correlates with attainment of this target when studying other beta-lactam antibiotics (as stated in *Introduction* page 3, line 82-84).

As there are other studies focusing on efficacy of cloxacillin, like the SNAP study, and much less is known about details regarding exposure of unbound cloxacillin, and its relation to renal function and toxicity, our main hypotheses are that i) renal function estimated by eGFR can predict which patients reach a target of 100% fT>MIC (i.e. 0.5 mg/l) and ii) there is a correlation between renal function, unbound plasma concentrations of cloxacillin and renal toxicity.

In order to more clearly explain the hypothesis to be tested and how this resulted in the sample size the section "*Hypothesis and sample size calculations*" has been rewritten. See page 8, lines 250-277.

3. How will you define what elevated urinary biomarker concentrations are?

RESPONSE: We thank the reviewer for this excellent question.

For U-cystatin C we will use the previously published reference interval: <0.414 mg/L. (Sohrabian A, Noraddin FH, Flodin M, Fredricsson A, Larsson A. Particle enhanced turbidimetric immunoassay for the determination of urine cystatin C on Cobas c501. Clin Biochem. 2012 Mar;45(4-5):339-44. doi: 10.1016/j.clinbiochem.2011.12.027.)

For U-alpha-1-microglobulin we will use the reference from the accredited hospital laboratory according to clinical routine: <10 mg/L.

U-KIM-1 and U-NGAL are less well standardized as there are no international recognised reference materials. The reference intervals should therefore be assay specific. We will use ELISAs from R&D Systems.

The decision limits for these assays will be:

U-KIM-1: <3530 pg/mL (According to the manufacturer, mean + 2SD (R&D Systems)

U-NGAL: <44 ng/mL (According to the manufacturer, mean + 2SD (R&D Systems)

The section Sample analysis (page 7, lines 202-206) has been adjusted to include information on how reference intervals are specified in the study.

Specific feedback:
The first sentence in abstract, introduction: Up to 30% of patients with ongoing antibiotic treatment do not survive Staphylococcus aureus bacteremia (SAB). This sentence doesn't make sense to me, what type of patients are you describing here (ie those that survive to step down or directed therapy)? This is higher than the usually quoted mortality rates for SAB.

RESPONSE: This is a valid comment as management of SAB has changed during the last decade. We have rewritten the sentence and updated it with a more recent publication where Swets et al references 90-day mortality data from 3 recent retrospective studies, without dividing the population into their suggested clinical subphenotypes. It now states: "*Staphylococcus aureus* bacteremia (SAB) is a common and severe infection, with a 90-day mortality of 24–32%."

Our aim is to describe the distribution of unbound cloxacillin exposure related to renal function and to explore if neurological- or renal damage is prevalent and associated with cloxacillin levels in real-world hospitalized SAB patients. – not sure distribution is the correct word here, maybe time-concentration profile, or temporal pharmacokinetics? Also, you aren't looking at the relationship of cloxacillin concentrations with kidney function in any endpoints. And this aim is only stated in the abstract.

RESPONSE: We thank the reviewer for finding and highlighting these discrepancies. We have revised and harmonized the aims in sections Abstract (page 1, lines 37-40) and in Introduction (page 3, lines 108-113).

In the introduction:
Renal function is a risk factor for insufficient exposure of beta-lactam antibiotics – specify augmented renal clearance is a risk factor for insufficient exposure of beta-lactam antibiotics.

RESPONSE: We appreciate this suggestion from the reviewer. We have adjusted the sentence in section Introduction (page 3, lines 81-82) accordingly.

Our aim is to describe the distribution of unbound cloxacillin exposure related to renal function and to explore if neurological- or renal damage is prevalent and associated with cloxacillin levels in real-world hospitalized SAB patients. – not sure distribution is the correct word here, maybe time-concentration profile, or temporal pharmacokinetics?

RESPONSE: The aim has been adjusted, see comments above.

Oxacillin beta-lactam antibiotics – should be anti-staphylococcal penicillins?

RESPONSE: We agree that the term needs clarification. We considered the suggestion from the reviewer but acknowledge that some *S. aureus* phenotypes show susceptibility to phenoxymethyl penicillins as well. In order to be as precise as possible, we change the term from *oxacillin beta-lactam antibiotics* to *isoxazolyl penicillins* throughout the manuscript.

Why would breastfeeding be interrupted?

RESPONSE: This is a valid question, as although the study is a clinical trial, it is open and builds rather on observation of standard procedures rather than any randomization or interventions being performed. However, the SMPC of cloxacillin states the following: “The drug is excreted in breast milk to a low degree. Effects on breastfed infants are unlikely, but effects on the infant's intestinal and oral flora cannot be excluded. Small amounts of the active substance in breast milk may increase the risk of sensitization.”

As of today > 100 individuals with SAB have been screened for the study and so far, no one has been excluded due to this specific criterion.

Were pregnant women included?

RESPONSE: As the SMPC of cloxacillin states that data on a large number of pregnancies does not indicate harmful effects on pregnancy or the health of the foetus/unborn child, we concluded that a pregnancy should not be an exclusion criterion. Thus, pregnant women will be included if the treating physician chooses to treat with cloxacillin, according to protocol.

Acute kidney injury is assessed by the change of serum creatinine levels, as described in the RIFLE criteria by Kidney Disease: Improving Global Outcomes (KDIGO) - RIFLE and KDIGO are separate.

RESPONSE: We thank the reviewer for noticing this discrepancy. We have revised the sentence that we use the KDIGO criteria, in accordance with the trial protocol (page 6, lines 186-187).

As a secondary endpoint: Descriptive characteristics of the SAB population – only SAB patients treated with cloxacillin, will this be the majority of SAB patients in all hospitals?

RESPONSE: This is a valid concern, since also flucloxacillin or cefazolin are considered first line treatments globally. However, neither flucloxacillin for parenteral use, nor cefazolin are marketed or sold in Sweden. Therefore, cloxacillin is considered first line treatment for patients with isolated MSSA SAB. Consequently, we expect that most patients will be treated with cloxacillin. However, there are clinical situations (ie. serious allergy, infection with other pathogens where a broader spectrum antibiotic is needed et.c) where some other antibiotic will be used for treatment. These situations are also exclusion criteria in the trial. We plan to register the reason for exclusion for all screened but not included individuals, to keep track of this.

VERSION 2 - REVIEW

**Reviewer**	**1**
**Name**	**Legg, Amy**
**Affiliation Department**	**Royal Brisbane and Women's Hospital, Pharmacy**
**Date**	**15-Mar-2026**

COI

Thank you, I accept you have addressed concerns to the best of your ability.
